# Phenotypic and genetic heterogeneity in a genome-wide linkage study of asthma families

**DOI:** 10.1186/1471-2466-5-1

**Published:** 2005-01-05

**Authors:** Janine Altmüller, Corinna Seidel, Young-Ae Lee, Sabine Loesgen, Dieter Bulle, Frank Friedrichs, Heidemarie Jellouschek, Julika Kelber, Angela Keller, Antje Schuster, Michael Silbermann, Wolfgang Wahlen, Peter Wolff, Gerhard Schlenvoigt, Franz Rüschendorf, Peter Nürnberg, Matthias Wjst

**Affiliations:** 1 gsf Institute of Epidemiology, GSF National Research Center for Environment and Health, Neuherberg, germany; 2 MDC Gene Mapping Center, Max-Delbrück Center for Molecular Medicine, Berlin, germany; 3 LoesGen, Oberbözberg, switzerland; 4 Praxis für Kinderheilkunde, Ravensburg, germany; 5 FF and K. Zima, Praxis für Kinderheilkunde, Aachen-Laurensberg, germany; 6 HJ and M. Barker, Klinik für Kinderheilkunde der RWTH Aachen, germany; 7 JK and W. Leupold, Klinik für Kinder und Jugendliche des Universitätsklinikums Carl Gustav Carus, Dresden, germany; 8 AK and W. Rebien, Praxis für Kinderheilkunde, Hamburg, germany; 9 Universitätskinderklinik, Düsseldorf, germany; 10 Praxis für Kinderheilkunde, Berlin, germany; 11 Praxis für Kinderheilkunde, Homburg, germany; 12 Praxis für Kinderheilkunde, Pfullendorf, germany; 13 Institut für Immunologie, Universität Jena, germany

## Abstract

**Background:**

Asthma is a complex genetic disease with more than 20 genome-wide scans conducted so far. Regions on almost every chromosome have been linked to asthma and several genes have been associated. However, most of these associations are weak and are still awaiting replication.

**Methods:**

In this study, we conducted a second-stage genome-wide scan with 408 microsatellite markers on 201 asthma-affected sib pair families and defined clinical subgroups to identify phenotype-genotype relations.

**Results:**

The lowest P value for asthma in the total sample was 0.003 on chromosome 11, while several of the clinical subsets reached lower significance levels than in the overall sample. Suggestive evidence for linkage (p = 0.0007) was found for total IgE on chromosomes 1, 7 and again on chromosome 11, as well as for HDM asthma on chromosome 12. Weaker linkage signals could be found on chromosomes 4 and 5 for early onset and HDM, and, newly described, on chromosome 2 for severe asthma and on chromosome 9 for hay fever.

**Conclusions:**

This phenotypic dissection underlines the importance of detailed clinical characterisations and the extreme genetic heterogeneity of asthma.

## Background

Many chromosomal regions have been shown to be linked or associated to asthma and asthma-associated traits in humans [1]. More recently, asthma genes have been identified on chromosomes 2 [2], 13 [3], 14 [4], and 20 [5]. The investigation of the genetic aetiology aims at the improvement of preventive strategies, diagnostic tools and therapeutic alternatives [6]. These final steps have not yet been reached or are even in sight, while the reasons for this delay are unclear.

The mainstay of all genetic studies has been genome-wide linkage scans in families with at least two asthma-affected siblings. Based on a previous analysis of a genome-wide scan of asthma [7,8] with inconsistent chromosomal findings to earlier studies, we decided to expand the initial sample with additional families by the same core protocol for clinical examination and using the same set of microsatellite markers [7,8]. The increased the number of identically pheno- and genotyped families could be used to define sub-phenotypes, which may be a promising strategy to explain the aetiological heterogeneity observed so far. Relevant clinical subsets may be defined by different age of onset, different disease course by degree of severity, extrinsic (allergic sensitization detectable) and intrinsic (no allergic sensitization detectable, symptoms often during infections of the upper respiratory tract) asthma type, and house dust mite allergy (HDM), as well as genetic background as judged by geographical origin of parents (table 1). We hypothesized that the restriction to a smaller well-defined sample would reduce heterogeneity and improve the power of detecting linkage. Linkage regions should show higher lod scores than in the total sample and lead from phenotype subgroups to a genotypic dissection.

**Table 1:** Phenotypes of the 201 families included.

**Phenotype**	**Definition**	**No. of families**
**Early onset asthma**	occurrence of asthmatic symptoms for one child before the age of 2 years and for his or her sib pair at least before the age of 4 years	67
**Extrinsic asthma**	all asthmatic children are positive for at least one SPT or specific IgE	134
**HDM SPT positive**	at least 3 family members with a positive skin prick test (SPT) for HDM	33
**HDM RAST positive**	at least 3 family members with a positive result of serum specific IgE for HDM	42
**Severe asthma**	asthma severity index (see table 3) for one asthmatic child is 4 or 5 and for his or her sib pair 3, 4 or 5	56
**Seasonal variation: **"Winter-Type"	one affected child suffers from asthma attacks in the winter half-year and his or her sib pair at least not solely in the summer half-year	39
**Seasonal variation: **"Summer-Type"	one affected child suffers from asthma attacks in the winter half-year and his or her sib pair at least not solely in the summer half-year	35
**German nationality**	both parents are of German descent	170

## Methods

### Clinical evaluation

97 families consisting of at least two children with confirmed clinical asthma were collected during the first stage of the German genome scan [7]. We now recruited another set of families during a period of 18 months. Trained staff from 3 university hospitals as well as 6 pediatric pulmonary practices carried out an identical phenotyping procedure as described previously [7]. This procedure contained detailed interviews of every family member, skin prick tests (SPT) of frequent allergens, blood samples (for IgE and allergen-specific IgE (RAST) measurements, eosinophil count), peak flow tests for a period of 10 days and dust collection at patients' homes. SPT and RAST assays included several pollens, animal furs, mould, and house dust mite allergens (ALK-SCHERAX, Hamburg, Germany). The ethics commission of "Nordrhein-Westfalen" approved all study methods and informed consent was obtained from all parents and children.

Children with premature birth, low birth weight and any severe pulmonary disease other than asthma were excluded as prematurity (and or associated low birth weight) may be a major non-genetic risk factor for the development of pulmonary symptoms. Another 4 families were excluded after genotyping because of Mendelian errors. The additional 104 families comprised 452 individuals (table 2). Their 244 children had a mean age of 10.8 years. 75 families contributed 2 children, 22 families 3 children and 7 families 4 children). In total, the 201 families had 465 children; 255 were male, 210 female, and 413 had physician-diagnosed asthma.

**Table 2:** Clinical characteristics of the 201 families (867 participants) that also include non-affected siblings of the core families. Descriptors of categorical variables include n/total sample in the first row and percent in the second row. Descriptors of continuous variables include mean values in the first row and standard deviation in the second row.

	**Parents**		**Children**	
	**Stage 1**	**Stage 2**	**Stage 1**	**Stage 2**

**sex females**	97/194 50.0%	104/208 50.0%	96/221 43.4%	114/244 46.7%
**Asthma diagnosis**	33/194 17.0%	60/208 28.8%	200/221 90.5%	213/244 87.3%
**D.pter skin > = 3 mm**	68/193 35.2%	58/206 28.2%	111/217 51.2%	107/242 44.2%
**D.far skin > = 3 mm**	57/193 29.5%	48/207 23.2%	101/217 46.5%	88/242 36.4%
**D.pter. CAP class >1**	51/183 27.9%	43/147 29.3%	124/217 57.1%	102/198 51.5%
**D.far. CAP class >1**	46/183 25.1%	39/147 26.5%	122/217 56.2%	100/198 50.5%
**age (years)**	39.8 +/-5.7	40.9 +/-5.5	11.0 +/-4.2	10.8 +/-3.4
**onset asthma (years)**	not reliable	not reliable	3.8 +/-2.8	4.3 +/-3.1
**height (cm)**	172.3 +/-9.0	172.3 +/- 9.9	146.9 +/-19.2	156.3 +/-18.1
**weight (kg)**	76.0 +/-14.9	78.0 +/-16.6	41.5 +/-17.0	47.4 +/-16.6
**ln(Ige) (kU/L)**	4.1 +/-1.7	4.1 +/-1.6	5.0 +/-2.0	5.0 +/-1.8
**ln(eosinophil) (count / mm3)**	0.3 +/-1.7	0.7 +/-1.5	1.1 +/-1.6	1.1 +/-1.6
**FEV1 (ml)**	3.594 +/-804	3.464 +/- 957	2.312 +/-883	2.861 +/-1.061

The trait "asthma" was based on the clinical diagnosis as described earlier [7]. "lnIgE" was used for analysis as a quantitative variable and categorized by cut-off point of 100 kU/l. Furthermore, the following phenotype subsets (see also table 1) were selected from the total study sample:

#### Early onset asthma

We created a subset of 67 families with one child having asthma symptoms before the age of 2 years and another child having asthma symptoms before the age of 4 years. In about half of the families within this subtype, both affected children had asthmatic symptoms before the age of 2 years. We were of course dependent on the recollection of the parents – but keeping in mind that the state of health of the children is of high priority in those families who joined our study and the period of time is quite clear, the data seemed valuable to us.

#### Extrinsic/intrinsic asthma sample

The group of families with atopic ("extrinsic") asthma was large, consisting of 134 families all with at least one positive skin prick test reaction or one increased specific IgE.

#### Positive Dermatophagoides farinae skin prick test and increased specific IgE in serum

Almost all family members participated in a skin prick test (SPT). We selected an "HDM SPT positive" group of 33 families with at least 3 family members showing a skin prick test reaction ≥ 3 mm. House dust mite allergy is quite common in asthmatic patients, and a known trigger factor for asthma attacks. Some studies have successfully used the combination with asthma as lead trait [9,10]. As the functional relevance might be slightly different in study participants with serum antibodies, another subgroup was based on the measurement of HDM-positive specific IgE at concentrations ≥ 0.35 kU/l, which resulted in a group of 42 families.

#### Asthma severity

Several interview questions related to asthma symptoms, diagnostic findings and therapy. We used information about asthma attack frequency, actual medication and emergency hospital visits for a severity index with 5 levels. This index is based on subjective patient information, current/previous therapy and influence on quality of life (table 3). "Severe Asthma" was seen in 56 families where one sib had at least grade 4 and another grade 3, 4 or 5. "Moderate Asthma" with one sib of maximum grade 2 and another with maximum grade 3 was defined in the same way.

**Table 3:** Asthma severity grade definition.

**Severity grade**	**Frequency and percentage in asthmatic children**	**Clinical criteria**		
		
		**Attacks (last 12 months)**	**Asthma medication (last 12 months)**	**At least 1 overnight hospital stay**
**1**	29 (7.2%)	none	none	none
**2**	118 (29.3%)	none	none	yes
		none	yes	none
**3**	164 (40.8%)	none	yes	yes
		once/month	yes	none
**4**	72 (17.9%)	once/month	yes	yes
		at least once/month	yes	none
**5**	19 (4.7%)	at least once/month	yes	yes

#### Seasonal variation of asthma attacks

All participants were asked to specify the months when asthma attacks occurred. 39 "winter-type" families had one affected child with symptoms only in the cold months and his or her sibs not a fully "summer-type". In contrast 35 "summer-type" families were found with one affected child with summer attacks and his or her sibs not purely "winter-type". Attacks year-round usually were seen with severe disease only. Sporadic attacks usually did not have any seasonal preferences while both the intrinsic, usually infection-related "winter-type", as well as the extrinsic, hay fever related "summer-type", appeared to be concordant within families. This observation may already support the hypothesis of different genes in different subtypes.

#### German sample

In 170 of 201 families, both parents were of German descent ("German"). In this subgroup, we tried to reduce the genetic heterogeneity by leaving out the remaining 31 families, of which 5 were from Sweden and 5 from Turkey.

### DNA analysis

DNA was isolated from peripheral white blood cells using the Puregene DNA isolation kit (Gentra Systems, Minneapolis, MN) according to the manufacturer's recommendation. For genotyping we used almost the same microsatellite marker as in the first scan [7,8]. The final analysis included 408 markers of which 364 were autosomal and 7 were based on the X-chromosome, all included in the previous scan as well as a set of 37 previous fine mapping markers. For the baseline marker set, the mean distance was 10 cM, with an average marker-information content of 0.87 and a mean heterozygosity of 0.79.

Marker amplification was performed in microtiter plates, either in 96- (Peltier Thermal Cycler PT-225, MJ Research, Waltham, MA) or in 384-well format (GeneAmp PCR System 9700, Applied Biosystems, Foster City, CA). Fragment analysis of PCR pools was conducted on an ABI 3700 DNA sequencer and genotypes were scored using GENESCAN and GENOTYPER (ABI) software. In the second scan, 98% of all possible genotypes could be unequivocally determined.

### Statistical analysis

Genotyping data were transferred to a SQL 2000 database, pre-checked with previously developed routines and exported in ASCII format for multipoint linkage analysis with MERLIN 0.9.3 [11]. Allele frequencies were estimated from the (unrelated) parental alleles. As different primers were used in some assays between the first scan in 1997 and the second scan in 2002, we adjusted the original allele size before replacing the allele size with its respective order. This procedure led to comparable allele frequency distributions in both scans. For ordering of all markers we used the Marshfield comprehensive human genetic linkage map that was slightly modified according to the marker order given by Golden Path 13 and expressed intermarker distances to Haldane cM. Error detection as implemented in MERLIN was then applied to discard unlikely genotypes from the analysis. Finally, P-values for qualitative traits were derived from the Kong and Cox method based on the score statistic S_all [12]. For the quantitative trait ln(IgE) a variance component was applied including only age and sex as covariates.

## Results

**Figure 1:**
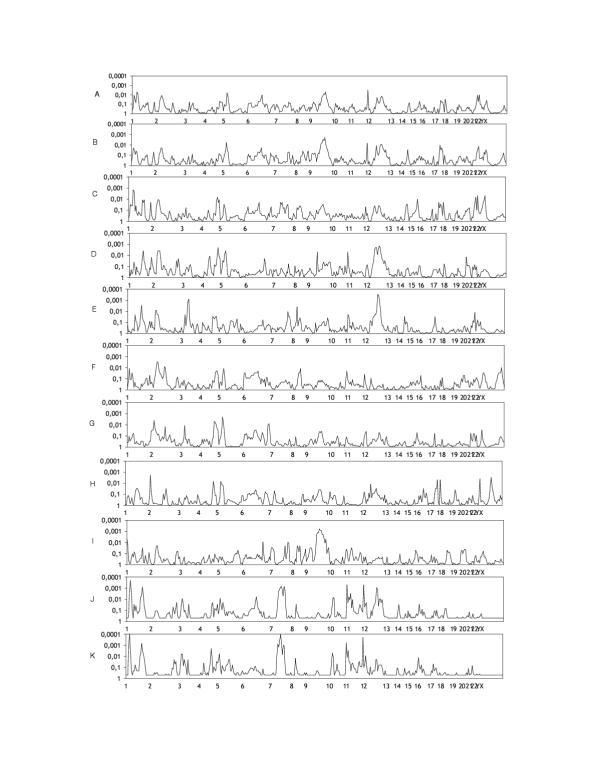
Multipoint linkage results for all traits. Chromosomes are arranged with increasing numbers with orientation from p-ter to q-ter. Distance is given in centimorgans (cM) on a linear scale. A = asthma all, B = asthma German families, C = extrinsic asthma, D = HDM RAST positive, E = HDM SPT positive, F = severe asthma, G = early onset asthma, H = winter-type, I = summer-type, J = LnIgE continuous, K = LnLgE categorical.

Figure 1 shows the linkage results for asthma, total IgE and all other subgroups.

Chromosomes are arranged by number from p-ter to q-ter with distance in centimorgans on a linear scale. Table 4 reports all loci with p-values below 0.01 in at least two adjacent markers for one phenotypic subgroup.

**Table 4:** Linkage results in 201 core families with at least 2 children having asthma. Weak linkage (p < 0.01) and suggestive linkage (p = 0.0007) is indicated in bold.

GDB	PIC	Chr.	cM (Marshfield)	Asthma All	Asthma German	Extrinsic Asthma	HDM RAST +ve	HDM SPT +ve	Severe Asthma	Early Onset	Winter-Type	Summer-Type	Ln(IgE) continuous	Ln(IgE) categorical
D1S478	0.8404	1	48.53	0.03	0.03	0.04	0.5	0.9	0.12	0.3	0.13	0.8	**0.002**	**0.0011**
D1S234	0.8559	1	55.1	0.07	0.08	0.04	0.3	0.9	0.12	0.2	0.2	0.8	**0.0002**	**0.00009**
D1S255	0.7710	1	65.47	**0.005**	0.04	**0.0015**	0.04	0.4	0.03	0.15	0.7	0.2	**0.002**	**0.005**
D1S197	0.8285	1	76.27	**0.007**	0.06	**0.002**	0.12	0.4	0.09	0.07	0.5	0.11	0.08	0.2

D1S484	0.8050	1	169.68	0.08	0.12	0.012	**0.004**	**0.003**	0.3	0.3	0.08	0.3	**0.007**	0.013
D1S431	0.9008	1	182.35	0.12	0.13	0.02	0.03	0.04	0.5	0.3	0.3	0.2	**0.005**	**0.005**
D1S2815	0.9201	1	188.85	0.06	0.09	0.012	0.06	0.07	0.3	0.3	0.2	0.03	**0.0008**	**0.0007**
D1S238	0.8722	1	202.73	0.4	0.7	0.4	0.2	0.1	0.3	0.8	0.2	0.6	**0.004**	**0.003**
D1S2655	0.8976	1	216.82	0.7	0.9	0.6	0.2	0.2	0.2	0.9	0.7	0.7	0.05	**0.007**

D2S2374	0.9039	2	54.96	0.03	0.07	0.04	0.011	**0.008**	0.04	0.02	0.8	0.4	0.3	0.5
D2S2328	0.9545	2	61	0.02	0.04	0.014	**0.007**	**0.007**	**0.008**	0.04	0.8	0.06	0.14	0.4
D2S2294	0.9617	2	64.84	0.02	0.03	0.02	**0.003**	0.02	**0.003**	0.5	0.8	0.02	0.13	0.4
D2S2298	0.9585	2	65.94	0.02	0.02	0.02	**0.003**	0.02	**0.002**	0.06	0.8	0.014	0.11	0.3
D2S2113	0.9331	2	88.15	0.3	0.5	0.3	0.2	0.4	**0.008**	0.13	0.7	0.3	0.5	0.5

D3S1597	0.8199	3	29.92	0.7	0.7	0.4	0.6	0.9	0.4	0.4	0.6	0.8	**0.009**	**0.006**
D3S1286	0.9021	3	41.56	0.4	0.3	0.3	0.2	0.3	0.13	0.2	1	0.4	0.02	**0.007**
D3S1300	0.8748	3	80.32	0.12	0.3	0.2	0.09	**0.0014**	0.3	0.5	0.3	0.8	0.4	0.09
D3S1285	0.7673	3	91.18	0.07	0.08	0.2	0.07	**0.0008**	0.2	0.2	0.13	0.6	0.5	0.5

D4S1607	0.8843	4	183.63	0.3	0.3	0.3	0.07	0.05	0.2	**0.005**	0.07	0.6	0.4	0.3
D4S1535	0.8938	4	195.06	0.05	0.07	0.013	**0.009**	0.03	0.03	**0.006**	**0.007**	0.11	0.07	0.09
D4S2924	0.8934	4	199.93	0.06	0.09	**0.007**	**0.002**	0.03	0.02	**0.009**	0.02	0.2	0.08	0.15

D5S426	0.8990	5	51.99	**0.006**	**0.006**	0.03	**0.008**	0.14	0.04	**0.002**	**0.007**	0.2	0.09	0.4
D5S418	0.9101	5	58.55	0.015	0.03	0.06	**0.004**	0.2	0.012	**0.007**	0.012	0.4	0.02	0.07

D7S484	0.9204	7	53.5	0.5	0.7	0.2	0.4	0.5	0.5	0.4	0.5	0.9	0.02	**0.009**
D7S528	0.9481	7	57.79	0.2	0.4	0.014	0.14	0.3	0.3	0.5	0.5	0.6	**0.0012**	**0.0007**
D7S510	0.9783	7	59.93	0.14	0.4	0.02	0.3	0.3	0.2	0.6	0.6	0.4	**0.0009**	**0.0013**
D7S485	0.9688	7	60.68	0.1	0.3	0.01	0.3	0.3	0.13	0.6	0.7	0.4	**0.0007**	**0.0004**
D7S2548	0.9596	7	62.28	0.2	0.4	0.05	0.4	0.5	0.12	0.7	0.7	0.3	**0.003**	**0.002**
D7S691	0.9403	7	63.67	0.08	0.2	0.02	0.4	0.5	0.08	0.6	0.6	0.3	**0.003**	**0.007**
D7S2506	0.9313	7	69.56	0.2	0.4	0.13	0.9	0.8	0.14	0.7	0.7	0.3	**0.007**	**0.011**
D7S663	0.9049	7	78.65	0.06	0.05	0.08	0.3	0.4	0.2	0.5	0.3	0.14	**0.0007**	**0.001**
D7S669	0.8351	7	90.42	0.08	0.05	0.04	0.4	0.2	0.13	0.5	0.5	0.02	**0.0014**	**0.0012**

D9S257	0.9630	9	91.87	0.09	0.06	0.3	0.3	0.4	0.4	0.4	0.4	**0.004**	0.5	0.5
D9S283	0.9681	9	94.85	0.015	0.01	0.1	0.2	0.3	0.2	0.11	0.1	**0.002**	0.2	0.3
D9S1796	0.9745	9	97.53	0.02	0.014	0.07	0.3	0.2	0.05	0.07	0.14	**0.006**	0.07	0.13
D9S1781	0.9565	9	99.4	0.015	**0.007**	0.05	0.14	0.2	0.11	0.03	0.03	**0.003**	0.07	0.15
D9S1851	0.9708	9	103.42	0.02	**0.008**	0.04	0.06	0.2	0.2	0.05	0.02	**0.003**	0.3	0.5
D9S1786	0.9825	9	104.48	0.013	**0.006**	0.03	0.04	0.11	0.13	0.05	0.02	**0.002**	0.4	0.5
D9S176	0.9798	9	105.02	0.02	**0.009**	0.05	0.09	0.1	0.2	0.14	0.03	**0.005**	0.5	0.5
D9S1690	0.9709	9	106.63	0.06	0.04	0.2	0.2	0.14	0.4	0.4	0.13	**0.007**	0.5	0.5
D9S1784	0.9667	9	111.99	0.11	0.04	0.2	0.07	0.2	0.3	0.6	0.4	**0.008**	0.5	0.5

D10S547	0.8313	10	29.15	0.14	0.2	0.4	0.8	0.8	0.6	0.3	0.2	0.9	**0.007**	**0.005**
D10S191	0.8726	10	37.9	0.4	0.5	0.2	0.3	0.5	0.8	0.5	0.3	0.8	**0.008**	0.012
D11S922	0.9045	11	2.11	0.2	0.4	0.4	0.3	0.02	0.13	0.3	0.9	0.06	**0.0005**	**0.0006**
D11S902	0.8133	11	21.47	0.3	0.3	0.5	0.9	0.3	0.3	0.5	0.6	0.5	**0.006**	**0.003**
D11S935	0.7997	11	45.94	0.5	0.4	0.4	0.4	0.4	0.4	0.6	0.9	0.05	**0.003**	0.04
D11S968	0.8094	11	147.77	**0.003**	**0.007**	0.05	0.11	0.3	0.04	0.12	0.6	0.2	**0.0005**	**0.0002**

D12S355	0.8343	12	74.58	0.07	0.08	0.06	**0.003**	0.04	0.4	0.2	0.012	0.6	0.3	0.4
D12S1684	0.9339	12	86.4	0.02	0.015	0.05	**0.002**	0.05	0.9	0.15	0.2	0.4	0.08	0.4
D12S1667	0.9727	12	92.89	0.05	0.08	0.2	**0.003**	0.04	0.8	0.15	0.08	0.4	0.03	0.2
D12S81	0.9707	12	94.44	0.08	0.12	0.2	**0.005**	0.02	0.8	0.3	0.09	0.3	0.013	0.2
D12S351	0.9822	12	95.56	0.02	0.013	0.05	**0.002**	**0.0003**	0.5	0.3	0.05	0.3	**0.0009**	0.02
D12S95	0.9785	12	96.09	0.012	**0.007**	0.04	**0.002**	**0.0002**	0.5	0.2	0.06	0.4	**0.0012**	0.02
D12S327	0.9757	12	97.78	0.02	**0.008**	0.07	**0.008**	**0.0009**	0.4	0.05	0.13	0.2	0.04	0.2
D12S1716	0.9590	12	101.45	0.04	**0.005**	0.2	0.02	**0.006**	0.4	0.06	0.2	0.2	0.011	0.06
D12S1706	0.9696	12	104.12	0.12	0.015	0.3	0.07	0.06	0.6	0.3	0.4	0.2	**0.007**	0.2

On chromosome 1, two linkage areas for total IgE could be found at about 55 cM (p = 0.00009) and at 188 cM (p = 0.0007), which reached the threshold for suggestive linkage (p = 0.0007) [13]. On chromosome 2 at about 64 cM (p = 0.002) evidence was found for a locus for severe asthma and for house dust mite sensitive asthma. Early onset, together with HDM, is represented both on chromosome 4 at about 195 cM (p = 0.002) and on chromosome 5 at about 55 cM (p = 0.002).

Three loci for total IgE regulation were found on chromosome 7 (65 cM, p = 0.0004) and chromosome 11 (2 cM, p = 0.0005; 147 cM, p = 0.0002), all with suggestive linkage according Kruglyak-Lander.

On chromosome 9 at about 104 cM (p = 0.002) there is evidence for a locus linked to allergic rhinitis plus asthma ("summer-type"), having an effect mainly in families of German descent. Lastly, a locus showing suggestive linkage could be identified on chromosome 12 (95 cM, p= 0.0002) for house dust mite sensitised asthmatic patients.

## Discussion

Several linkage regions in asthma families could be found in this extended sample, although again no linkage with P < 0.003 could be found with any marker for asthma. Asthma is apparently of such a heterogeneity that even investigating affected sib pairs from more than 200 families failed to yield significant results. Nevertheless, the quantitative trait IgE in these families, both as a discrete and continuous variable, led to three suggestive linkage findings on chromosomes 1, 7, and 11, which have all been discussed in prior publications (table 5).

**Table 5:** Comparison of asthma loci

Chr. Position	This study (2004)	Bradley (2002) [14]	Cookson (2001)[15]	Daniels (1996) [16]	Dizier (2000) [17]	Laitinen (2001) [18]	Malerba (1999) [19]	Ober (1998. 1999, 2000) [20-22]	Xu J (2000, 2001) [23, 24]	Xu X (2001) [25]	Yokouchi (2000) [9]	CSGA (1997) [26]
**1 **55cM	IgE				Asthma			Asthma, Atopy	Asthma Hispanic population			

**1 **188cM	IgE		AD		Slope					IgE		

**2 **64cM	severe asthma HDM											

**3 **50cM	IgE HDM	AD						Loose Asthma				

**4 **195cM	early onset HDM			Slope							HDM and Asthma	

**5 **55cM	early onset HDM				Slope			BHR				Asthma

**7 **65cM	IgE			IgE, Slope, Eosinophils	SPT	Asthma, IgE						

**9 **104cM	German summer type							Asthma symptoms, Atopy				

**11 **2cM	IgE				IgE			SPT	Asthma Hispanic population			Asthma

**11 **147cM	IgE				IgE							

**12 **95cM	HDM				Eosino-phils, IgE		Asthma	Asthma	IgE, Asthma		HDM and Asthma	Asthma

### Phenotypic dissection

Moreover, the strategy of phenotypic dissection proved to be quite successful, though the sample size of subsets seems to be very small. The size of the sample in a linkage study is known to be one of the most important factors for a significant finding [1], making the artificial limitation of subsets a double-edged decision. Clearly, there is a remarkable phenotypic heterogeneity of asthmatic diseases but – as these families represent different genetic influences-limitation of the sample size did not affect statistical properties as significance values were smaller compared to the whole study sample.

In particular, the subgroups segregated by high disease severity, sensitisation by HDM, early onset and population origin attained better lower significance levels than the main trait (table 4). So far, results were not adjusted with the Bonferroni correction for multiple testing as this would have been too conservative for this explorative investigation. Subgroups are often closely related and highly overlapping. Nevertheless, there might be a problem with multiple testing and the criteria for suggestive linkage in a genome scan on a single phenotype [13].

Sample recruitment with restricted phenotypic requirements proved to be successful for some other traits as well. Early onset definitions have been used for cancer studies and metabolic disorders to increase the underlying genetic component [27,28]. Separation in sub-samples of ethnically diverse populations has also been conducted [26], but higher disease activity scores [29] seemed to give an advantage in other fields also.

### Other Asthma Genome Scans

Recently, evidence was shown for asthma genes on chromosomes 2 [2], 13 [3], 14 [4], and 20 [5]. Our study does not support linkage loci in those regions. As the region on chromosome 13 was responsible for only 10% of the observed IgE serum level variability, this small effect may be easily overlooked in another study. A lack of replication does not necessarily mean an error in the primary study. Most likely, genetic heterogeneity is the cause, where the proportion of patients with a particular gene variant is different between studies. A polygenic model of the inheritance mode of asthma with minor effects by single genes could explain the highly diverse association and linkage results thus far.

Frequent replication, however, may point toward lead genes. Most regions listed in table 4 have already been described and some of the traits are supported by the phenotypes proposed in our analysis. Table 5 summarizes previously published asthma/atopy loci in approx. 20 cM distance that would be consistent with our findings. Both loci on chromosomes 4 and 5 are supported by the phenotypes of early onset, HDM and "winter-type", which might relate to the clinical picture of chronic obstructive bronchitis. Phenotypes described by other studies include lung function variation and HDM, which is supported by our data. The "atopy" locus (defined by SPT and RAST) on chromosome 3 confirms linkage studies of atopic dermatitis (AD) [14] and might be the genetic link between both diseases.

The locus on chromosome 12 with suggestive linkage for HDM in our study is known from many other reports. Also our first scan showed weak evidence for linkage in this region at D12S351 (p = 0.01) [7], whereas the best result for the HDM subset lies now about 0.5 cM apart at D12S95 with p = 0.0002. These consistent findings are probably based on frequent alleles playing an important role in extrinsic asthma. Linkage results for SPT and RAST for HDM were quite similar, but the HDM SPT positive group – which is a smaller group-, reached the suggestive linkage level on chromosome 12. SPT, even more than RAST, might represent the individual's allergic disposition.

On chromosome 2, we found a locus in families with severe asthma that has not yet been described in other studies. A p-value of 0.007 has already been found in our first genome-wide scan [7] for D2S2298 and our main trait asthma. The same marker for severe asthma in both samples improved to a p-value of 0.002. Severe asthma is rather rare, and a disease-aggravating allele on chromosome 2 could be easily overlooked in a heterogeneous asthma sample. Severe asthma may be a lethal disease, while a severe-asthma gene would be of high importance for intensified treatment.

The German "summer-type" locus on chromosome 9 is also not frequent in other studies. This might be due to the fact that many, but not all, genes are necessary for a common trait. Our first asthma scan has already shown linkage in this region with a p-value of 0.007 for D9S1784 [7], which now improves to p = 0.002 for the "summer-type" subset at D9S1786, about 7.5 cM apart.

## Conclusions

We conclude that this phenotypic dissection is a useful tool to detect linkage in a heterogeneous disease like asthma because some of the sub phenotypes reached even better significance values than the main trait. We show that the precision of the phenotype can be more effective than expanding the sample size only. Unfortunately, large sample sizes are needed to assure at least moderate sample size in subsets. Grouping by early onset or disease severity could be applied to almost every complex disease, but for a more specific dissection, clinical expert knowledge is required. A prior analysis of clinical data can help to identify symptom clustering, which -if consistent within families- can reduce genetic heterogeneity.

## Competing interests

The author(s) delcare that they have no competing interests.

## Authors' contributions

MW, JA, and CS were involved in the study design, JA organised the sample collection, conducted the genetic analysis, and prepared the manuscript, MW organized funding and supervised the study. YAL and PN organised and helped with genotyping and revised the article critically for important intellectual content. SL, FR and MW carried out the statistical analysis and made substantial contributions to its conception and design, CS, DB, FF, HJ, JK, AK, AS, MS, WW, PW assisted in the recruitment of families and examined patients and therefore made substantial contributions to the acquisition of data, asthma severity grades are part of the thesis of CS. GS was responsible for the IgE analysis.

## Pre-publication history

The pre-publication history for this paper can be accessed here:


